# Mental health and gestational weight gain: A comparison between Brazilian cohorts

**DOI:** 10.1371/journal.pone.0326743

**Published:** 2025-08-04

**Authors:** Audêncio Victor, Maria Paula Carvalho Leitão, Perla Pizzi Argentato, Lívia Patricia Rodrigues Batista, Laisla de França da Silva Teles, Liania A. Luzia, Rinaldo Artes, Patricia H. C. Rondó

**Affiliations:** 1 Public Health Postgraduate Program, School of Public Health, University of São Paulo, Rua Av. Dr. Arnaldo, 715 - Cerqueira César, São Paulo - SP, Brasil; 2 Nutrition Department, School of Public Health, University of São Paulo, Rua Av. Dr. Arnaldo, 715 - Cerqueira César, São Paulo - SP, Brasil; 3 Insper - Institute of Education and Research, Rua Quatá, 300, São Paulo - SP, Brazil; King Abdulaziz University Faculty of Medicine, SAUDI ARABIA

## Abstract

**Introduction:**

The mental health of pregnant women is critical as it influences both maternal and neonatal outcomes. This study investigates the association between maternal mental health and gestational weight gain (GWG) in two Brazilian cohorts conducted in different periods.

**Methods:**

The Jundiaí cohort (1997–2000) included 875 pregnant women, while the Araraquara cohort (2017–2024) evaluated mental health of 556 pregnant women from 2017 to 2019. Maternal mental health was assessed using the General Health Questionnaire (GHQ), the State-Trait Anxiety Inventory (STAI), and the Perceived Stress Scale (PSS) during the first, second, and third trimesters. GWG was categorized as adequate, insufficient, or excessive based on Institute of Medicine guidelines. Statistical analysis included bivariate tests (Kruskal-Wallis, chi-square, or Fisher’s exact test) and multinomial ordinal logistic regression to evaluate associations.

**Results:**

In the Jundiaí cohort, high stress levels in the first trimester were associated with lower odds of insufficient GWG (adjusted OR for second quartile: 0.36, 95% CI: 0.18–0.71). In the second trimester, high anxiety levels (TAI ≥ 40) were associated with higher odds of insufficient GWG (ORa: 1.76, 95% CI: 1.12–2.76). In the third trimester, high stress levels (PSS fourth quartile) were associated with higher odds of insufficient GWG (adjusted OR: 1.72, 95% CI: 1.02–2.91). In the Araraquara cohort, no significant associations between mental health and GWG were found.

**Conclusions:**

Our findings highlight the importance of incorporating psychosocial support in prenatal care to improve maternal and neonatal outcomes. Variations in socioeconomic and temporal contexts may influence the relationship between mental health and GWG. Future research should explore the underlying mechanisms and develop interventions tailored to different socioeconomic and temporal contexts.

## Introduction

Pregnancy is a critical period in a woman’s life marked by significant physiological and psychological changes [1,2], often accompanied by mental health disorders such as depression and anxiety [3,4]. A recent systematic review showed global rates of depression during pregnancy ranging from 15% to 65% [5]. In Western countries, 5% to 20% of pregnant women experience psychological disorders [3,4,6]. In low- and middle-income countries, this prevalence may be higher [4,7]. Studies in Brazil have shown prevalence rates of about 14% to 27% for depression in pregnant women [8–11] and 19% to 40% for anxiety [12–14].

Gestational weight gain (GWG) is a crucial aspect of pregnancy, directly influencing maternal and fetal health [15–18]. Excessive GWG is associated with increased risks of obstetric complications, including gestational diabetes, preeclampsia, cesarean section, macrosomia, metabolic diseases, and cardiometabolic diseases in childhood [16,19–22]. On the other hand, insufficient GWG is associated with low birth weight, preterm birth, intrauterine growth restriction, and perinatal mortality [23–25]. Maternal mental health plays a key role in determining GWG, as conditions such as depression, anxiety, and stress can negatively influence eating habits, physical activity levels, and metabolic regulation during pregnancy [12,26]. Psychological distress can lead to inadequate dietary intake, emotional eating, and altered hormonal responses, all of which can affect GWG patterns. Current literature indicates a complex and significant interconnection between mental health and GWG, with some studies reporting that maternal mental health disorders are associated with both insufficient and excessive GWG, depending on the severity and nature of the condition [8,9,12]. This situation underscores the imperative need for greater attention and investigation on this topic [2].

In Brazil, socioeconomic disparities and unequal access to healthcare can further complicate this relationship, as lower-income women may have limited access to mental health services and nutritional guidance during pregnancy [27]. Over the past two decades, improvements in prenatal care and increased mental health awareness may have influenced the association between maternal mental health and GWG [9,12,27]. However, there is limited evidence on how these changes have impacted different socioeconomic groups.

Maternal mental health during pregnancy can play a role in determining GWG [8,9]. However, study results are still inconclusive [8,28,29]. Moreover, the effect of these disorders on GWG trajectories remains uncertain, especially in low- and middle-income countries, where the prevalence of mental health problems has substantially increased [4,7]. Analysing two Brazilian cohorts enables us to assess if shifts in socioeconomic conditions and access to prenatal healthcare have affected the link between maternal mental health and GWG. By integrating validated mental health assessment tools, such as the General Health Questionnaire (GHQ), the State-Trait Anxiety Inventory (STAI), and the Perceived Stress Scale (PSS), this study ensures a robust evaluation of maternal psychological distress and its potential effects on GWG. With this, we hope to contribute to improving health outcomes for mothers and children, emphasizing the importance of addressing mental health as a key factor in reducing gender health disparities.

## Methods

### Study design

This is an observational epidemiological study of the prospective cohort type, comparing two cohorts separated by a 20-year interval, respectively, in two municipalities with similar socioeconomic characteristics in the state of São Paulo, Brazil:

The Araraquara cohort study: The sample included women with gestational age ≤ 19 weeks who received prenatal care in the 37 Health Units and at the Special Health Service (SESA) in the municipality of Araraquara, São Paulo, Brazil. Pregnant women participating in the study were followed quarterly throughout prenatal care until the birth of their children from 05 January 2017–30 December 2024 at the “Gota de Leite” Municipal Maternity. All participants were users of the Brazilian Unified Health System (SUS), a universal public healthcare system that serves the entire population [30,31]. Women with twin pregnancies and those who had abortions were excluded. Pregnant women with missing information on height, pre-gestational weight, and weight at the date of delivery were also excluded [32,33].

Jundiaí cohort study (USP-MatStress): From an initial sample of 1182 women who received prenatal care between 03 June 1998 and 20 August 2000 in 12 health units and five hospitals in the Municipality of Jundiaí, São Paulo, Brazil, 865 were followed in a cohort study from before the 16th week of pregnancy until the birth of their children [34]. The study conducted in Araraquara was approved by the Research Ethics Committee with Human Subjects at the School of Public Health, University of São Paulo, before data collection under protocol number CAEE: 59787216.2.0000.5421, with opinion number 1.885.874. The study conducted in Jundiaí was also approved, protocol number 289/98 and informed written consent was obtained from all participants. The protocol was approved by the Ethical Committees of the School of Public Health, University of São Paulo, and the Health Secretariat of Jundiaí, SP.

### Outcome

Weekly average GWG during the second and third trimesters of pregnancy was assessed according to National Academy of Medicine guidelines, being classified as adequate, insufficient, or excessive. The adequate GWG category was used as a reference for comparison with other categories. Pre-gestational BMI was determined by considering the weight and height before pregnancy, allowing the classification of adequate weekly GWG during the second and third trimesters as recommended by the National Academy of Medicine [35]. Pre-gestational BMI categorization followed the criteria of the World Health Organization [36].For women who start pregnancy with a pre-gestational BMI < 18.5 kg/m2, a weight gain of 440–580 grams per week is recommended. Those with a BMI of 18.5 to 25.0 kg/m2 should gain 350 to 500g, a BMI of 25–30 kg/m2 should gain 230 to 330g, and with a BMI ≥ 30.0 kg/m2, the recommended range is 170 to 270g. The average GWG during the second and third trimesters was calculated by the weight difference between the last prenatal visit of the third trimester and the first visit of the second trimester divided by the number of weeks between these visits. This allowed the classification of pregnant women’s GWG as adequate, insufficient, or excessive [35]. Regarding weight measurements, the weight of pregnant women was directly measured during study visits, ensuring accuracy.

### Main exposure

#### Mental health during pregnancy.

Three standardized questionnaires were used to assess pregnant women’s mental health, including the GHQ, STAI, and the PSS.

### General mental health

Maternal psychological distress was assessed using the 12-item version of the GHQ [37]. This questionnaire is a screening tool designed for use in research with general populations. Scores indicate the severity of psychological distress on a continuum. The validity of this questionnaire has been tested in a Brazilian population [38] including pregnant women [14,34]. According to GHQ scores, pregnant women were classified into two groups: low (0–3) and high (≥ 4) [14,37,38].

### Anxiety

Maternal anxiety, both state and trait, was assessed using the STAI. The STAI is a well-standardized self-report instrument with 40 items designed to measure anxiety at the time of the interview (state-SAI) and anxiety in the last month (trait-TAI). State and trait anxiety were assessed using a cut-off point of ≥ 40 in scores used in Brazilian studies with pregnant women [14,34].

### Stress

Maternal stress was assessed using the 14-item PSS [39].This scale determines the extent to which situations in the past 30 days were perceived as stressful. The PSS was designed for use in community samples due to its ease of understanding and simple responses on a five-point scale ranging from ‘never’ to ‘very often.’ Since there is no clear cut-off point for PSS scoring, results were analyzed in quartiles [14]. This version has been validated for use in the Brazilian population [40].

### Covariates

#### Maternal characteristics.

Several factors were considered as covariates for the study, including socioeconomic and demographic characteristics. These included age (≤ 19, 20–35, and > 35 years), education level (≤ 12 and > 12 years), number of people per room, per capita income in Brazilian reais (1 US$ = 4.9 R$), race (white or non-white), and marital status (married/stable union, single/separated/widowed). Lifestyle habits included physical activity, smoking, and alcohol consumption. Anthropometric variables examined were BMI (kg/m²), arm circumference (cm), number of previous pregnancies (categorized as 1 and less and 2 and more), and hemoglobin (g/dL).

### Theoretical model

This study is guided by a theoretical model that examines the relationship between maternal mental health and GWG in two Brazilian cohorts. Maternal psychological well-being during pregnancy can influence dietary habits, metabolic processes, and overall weight gain. Psychological distress including stress, anxiety, and depression may lead to altered eating behaviours, reduced or excessive food intake, and metabolic imbalances that impact GWG [4,26]. Moderating factors such as socioeconomic conditions, access to prenatal care, and mental health support may shape this relationship [27,41]. Mental health was assessed using validated instruments (GHQ, STAI, and PSS), and GWG was classified as insufficient, adequate, or excessive based on the National Academy of Medicine guidelines. [42].

### Statistical analysis

Descriptive statistics were used for data analysis. The Shapiro-Wilk test was applied to assess the normality of continuous variables. Normally distributed variables were reported as mean (SD), while non-normal continuous variables were presented as median (IQR). Bivariate analyses were conducted to examine associations between mental health and GWG. For continuous variables, the Kruskal-Wallis test was used, while for categorical variables, the chi-square or Fisher’s exact test was employed when normality was not met. Data modeling was conducted using ordinal multinomial logistic regression analysis, allowing the assessment of associations between multiple variables related to mental health and a dependent variable with three or more ordered categories, such as the categories of GWG adequacy according to National Academy of Medicine guidelines [43]. Model adjustment was performed following a step-by-step strategy, an iterative method that selects and removes independent variables based on statistical criteria. Variables were kept in the model if they presented a significance level of p < 0.02. During the adjustment process, variables from the initial theoretical model were considered. Results were presented in measures of association (OR) with statistical significance indicated by P < 0.05 and 95% confidence intervals. All analyses were conducted using R software (version 4.1.0, R Foundation for Statistical Computing, Vienna, Austria).

## Results

### Maternal characteristics and GWG in two cohorts

According to the data presented in Table 1, 875 women in the Jundiaí cohort and 556 in the Araraquara cohort were evaluated. The predominant age group in both cohorts was 20–35 years, representing 657 (75.3%) women in Jundiaí and 423 (76.1%) in Araraquara, maternal education revealed in Jundiaí, 847 (97.1%) women had up to 12 years of education, a significant contrast with Araraquara, where this number was 481 (86.5%). Regarding lifestyle, 120 (14.0%) were smokers in Jundiaí, while in Araraquara, this number was 44 (7.9%). In terms of alcohol consumption, 189 (22.0%) were reported in Jundiaí and 121 (21.8%) in Araraquara. The median pre-gestational BMI was 23.0 kg/m² (21.1–25.8) for Jundiaí and 25.3 kg/m² (21.8–29.2) for Araraquara. Additionally, the average arm circumference was 26.9 cm (24.9–29.0) in Jundiaí versus 29.4 cm (26.5–32.5) in Araraquara. The median hemoglobin level was 12.6 g/dL (11.9–13.3) in Jundiaí and 12.5 g/dL (12.0–13.1) in Araraquara. For socioeconomic status, the number of people per room was categorized into tertiles, in Jundiaí, 325 (37.3%) of women were in the first tertile (lowest crowding), 416 (47.7%) in the second tertile, and 131 (15.0%) in the third tertile (highest crowding). In Araraquara, the distribution was 256 (46.0%) in the first tertile, 113 (20.3%) in the second tertile, and 184 (33.2%) in the third tertile,

**Table 1:** Characteristics of pregnant women in Araraquara and Jundiai Cohort Studies, São Paulo, Brazil.

Variables	Cohorts		P value
	Jundiai (n = 875)	Araraquara (n = 556)	
**Age (year)**			
≤19	155 (17.8)	68 (11.7)	
20-35	657(75.3)	423 (76.1)	0.0004
>35	60 (6,9)	65 (11.7)	
**Education (years)**			
≤12	847 (97.1)	481 (86.5)	<0.0001
>12	25 (2.9)	75 (13.5)	
**Number of people per room (tertile)**			
1st	325 (37.3)	256 (46.0)	
2nd	416 (47.7)	113 (20.3)	<0.0001
3rd	131 (15.0)	184 (33.2)	
**Race**			
White	714(18.1)	261 (46.9)	<0.0001
Non white	158 (81.9)	295 (53.1)	
**Marital status**			
Married or in a stable relationship	672 (77.1)	500 (89.9)	<0.0001
Single, separated, or widowed	200 (22.9)	56 (10.1)	
**Smoking**			
No	738 (86.0)	512(92.1)	0.0007
Yes	120 (14.0)	44 (7.9)	
**Alcohol consumption**			
No	669 (78.0)	435 (78.2)	0.9585
Yes	189 (22.0)	121 (21.8)	
**Pre-gestational BMI (kg/m²)**	23.0 (21.1-25.8)	25.3 (21.8-29.2)	<0.0001
**Arm circumference (cm)**	26.9 (24.9-29.0)	29.4 (26.5-32.5)	<0.0001
**Number of previous pregnancies**			
1 and less	483 (55.4)	397 (28.6)	<0.0001
2 and more	389 (44.6)	159 (71.4)	
**Hemoglobin (mg/dL)**	12.6 (11.9-13.3)	12.5 (12-13.1)	0.0005
**GWG during second and third trimesters**			
Adequate	197 (22.6)	107 (19.2)	0.0029
Excessive	420 (48.2)	319 (57.4)	
Insufficient	255 (29.2)	130 (23.6)	

Regarding reproductive history, 483 (55.4%) women in Jundiaí reported having had one or fewer previous pregnancies, while in Araraquara, this percentage was 397 (71.4%). Regarding GWG, the Araraquara cohort had a higher incidence of excessive GWG, with 319 (57.4%) compared to 420 (48.2%) in the Jundiaí cohort. Conversely, adequate GWG was recorded in 197 (22.6%) women in Jundiaí and 107 (19.2%) in Araraquara. Statistically significant differences were observed between cohorts for most variables, including maternal age (p = 0.0004), education level (p < 0.0001), number of people per room (p < 0.0001), race (p < 0.0001), marital status (p < 0.0001), smoking (p = 0.0096), alcohol consumption (p = 0.5095), number of previous pregnancies (p < 0.0001), and gestational weight gain category (p = 0.0006).

Significant differences were also found for clinical variables: pre-gestational BMI (p < 0.0001), arm circumference (p < 0.0001), and hemoglobin concentration (p = 0.0005).

**Table 2** shows that in the first trimester, the PSS indicated a significant difference in Jundiaí (P = 0.005), where women in the 1st quartile (indicative of very low stress) had a lower incidence of excessive GWG (13.8%) compared to those in the 3rd quartile (indicative of high stress) with 14.9%. In the second trimester, a statistically significant difference (P = 0.04) was observed in the GHQ in Jundiaí, where 36.5% of women with low scores (fewer psychological symptoms) had excessive GWG. The TAI showed a significant difference in Araraquara (P = 0.04), with 32.7% of women having low anxiety and excessive GWG. In the third trimester, no significant differences were found in any of the mental health instruments evaluated concerning GWG in both cohorts.

**Table 2:** Mental health and its relationship to gestational weight gain in the first, second, and third trimesters in Araraquara and Jundiaí Cohort Studies, São Paulo, Brazil.

Variables		Cohorts studies							
		Jundiai			P values	Araraquara			P values
			Adequate	Excessive	Insufficient	Adequate	Excessive	Insufficient	
1st Trimester	**General Health Questionnaire**								
	≤ 3 (Low)	142 (16.6)	282 (32.9)	161 (18.8)	0.22	50 (10.0)	159 (31.9)	51 (10.2)	0.12
		4–12 (High)	54 (6.3)	131 (15.3)	88 (10.2)		44 (8.8)	129 (25.9)	65 (13.1)
	**Trait Anxiety Inventory (TAI)**								
	< 40 (Low Anxiety)	89 (10.2)	185 (21.2)	111 (12.7)	0.94	50 (8.9)	150 (27.0)	48 (8.6)	0.13
		≥ 40 (High Anxiety)	108 (12.4)	235 (26.9)	144 (16.5)		57(10.3)	169 (30.4)	82 (14.7)
	**State Anxiety Inventory (SAI)**								
	< 40 (Low Anxiety)	132 (15.1)	279 (31.9)	156 (17.8)	0.31	60 (10.8)	162 (29.1)	71 (12.8)	0.56
		≥ 40 (High Anxiety)	65 (7.5)	141 (16.2)	99 (11.4)		47 (8.4)	157 (28.2)	59 (10.6)
	**Perceived Stress Scale (PSS)**								
	1st quartile (Very Low)	32 (5.7)	78 (13.8)	52 (9.3)	**0.005**	21 (4.2)	69 (13.8)	23 (4.6)	0.75
		2nd quartile (Low)	39 (6,9)	60 (10.7)	24 (4.3)		19 (3.8)	75 (15.0)	27 (5.4)
		3rd quartile (High)	40 (7.1)	84 (14.9)	32 (5.7)		20 (4.0)	51 (10.2)	20 (4.0)
		4th quartile (Very High)	20 (3.6)	57 (10.1)	44 (7.8)		34 (6.8)	94 (18.8)	46 (9.2)
2nd Trimester	**General Health Questionnaire**								
	≤ 3 (Low)	154 (17.9)	313 (36.5)	171 (19.9)	**0.04**	63 (12.7)	192 (38.8)	67 (13.5)	0.34
		4–12 (High)	42 (4.9)	100 (11.7)	78 (9.1)		32 (6.4)	95 (19.2)	46 (9.3)
	**Trait Anxiety Inventory (TAI)**								
	< 40 (Low Anxiety)	113 (12.9)	214 (24.5)	126(14.8)	0.21	53 (9.5)	182(32.7)	58(10.4)	**0.04**
		≥ 40 (High Anxiety)	84 (9.6)	206 (23.6)	129 (14.8)		54 (9.7)	137 (24.6)	72 (12.9)
	**State Anxiety Inventory (SAI)**								
	< 40 (Low Anxiety)	144 (16.5)	298 (34.2)	175 (20.1)	0.58	69 (12.4)	199 (35.8)	71 (12.8)	0.22
		≥ 40 (High Anxiety)	53 (6.1)	122 (14.0)	80 (9.2)		38 (6.8)	120 (21.6)	59 (10.6)
	**Perceived Stress Scale (PSS)**								
	1st quartile (Very Low)	52 (6.1)	110 (12.8)	69 (8.0)	0.07	23 (4.6)	86 (17.4)	24 (4.8)	0.17
		2nd quartile (Low)	64 (7.5)	110 (12.8)	63 (7.3)		27 (5.5)	63 (12.7)	27 (5.5)
		3rd quartile (High)	47 (5.5)	87 (10.1)	44 (5.1)		25 (5.1)	76 (15.4)	25 (5.1)
		4th quartile (Very High)	33 (3.8)	106 (12.4)	73 (8.5)		20 (4.0)	62 (12.5)	37 (7.5)
3rd Trimester	**General Health Questionnaire**								
	≤ 3 (Low)	154 (17.9)	302 (35.2)	171 (19.9)	0.07	64 (13.1)	196 (40.0)	71 (14.5)	0.46
		4–12 (High)	42 (4.9)	111 (12.9)	78 (9.1)		28 (5.7)	89 (18.2)	42 (8.6)
	**Trait Anxiety Inventory (TAI)**								
	< 40 (Low Anxiety)	110 (12.6)	224 (25.6)	133 (15.3)	0.73	61 (8.3)	199 (35.8)	70 (12.5)	0.21
		≥ 40 (High Anxiety)	87 (9.9)	196 (22.5)	122 (13.9)		46 (8.3)	120 (21.6)	60 (10.8)
	**State Anxiety Inventory (SAI)**								
	< 40 (Low Anxiety)	135 (15.5)	284 (32.6)	168 (19.3)	0.82	72 (12.9)	197 (35.4)	78 (14.0)	0.48
		≥ 40 (High Anxiety)	62 (7.1)	136 (15.6)	87 (9.9)		35 (6.3)	122 (21.9)	52 (9.4)
	**Perceived Stress Scale (PSS)**								
	1st quartile (Very Low)	60 (6.9)	113 (13.2)	74 (8.6)	0.92	28 (4.6)	79 (17.4)	27 (4.8)	0.66
		2nd quartile (Low)	47 (5.5)	94 (11.0)	54 (6.3)		19 (5.5)	83 (12.7)	29 (5.5)
		3rd quartile (High)	52 (6.1)	112 (13.1)	64 (7.4)		22 (5.1)	60 (15.4)	26 (5.1)
		4th quartile (Very High)	37 (4.3)	106 (10.9)	57 (6.6)		23 (4.0)	63 (12.5)	31 (7.5)

### Association between mental health and GWG

As shown in Table 3, in the Jundiaí cohort after adjustments, the second trimester showed that women with high scores (4–12) on the GHQ had a significantly higher chance of insufficient GWG (OR 1.76, 95% CI 1.12–2.76), while women in the second quartile (low) of PSS had a lower chance of insufficient GWG (adjusted OR 0.36, 95% CI 0.18–0.71). In the third trimester, the same level of stress was still significantly associated with insufficient GWG (OR 1.58, 95% CI 1.01–2.47). No significant associations between mental health scores and GWG were found in the Araraquara cohort.

**Table 3:** Crude and adjusted ordinal multinomial logistic regression analyses between mental health and gestational weight gain in the first, second, and third trimesters in Araraquara and Jundiai Cohort Studies, São Paulo, Brazil.

Variables		Cohort studies							
		Jundiai				Araraquara			
		Crude Model		Adjusted Model		Crude Model		Adjusted Model	
		Excessive	Insufficient	Excessive	Insufficient	Excessive	Insufficient	Excessive	Insufficient
		OR (IC95%)	OR (IC95%)	ORa (IC95%)	ORa (IC95%)	OR (IC95%)	OR (IC95%)	ORa (IC95%)	ORa (IC95%)
1st Trimester	**General Health Questionnaire**								
	≤ 3 (Low)	1	1	1	1	1	1	1	1
	4–12 (High)	1.22 (0.84-1.78)	1.44 (0.96-2.16)	1.36 (0.92-2.03)	1.46 (0.95-2.23)	0.92 (0.58-1.47)	1.45 (0.84 - 2.5)	0.95 (0.57-1.58)	1.56 (0.87-2.78)
	**Trait Anxiety Inventory (TAI)**								
	< 40 (Low Anxiety)	1	1	1	1	1	1	1	1
	≥ 40 (High Anxiety)	1.05 (0.29-1.39)	1.07 (0.31-1.44)	1.25 (0.14-1.59)	1.14 (0.26-1.53)	0.99 (0.45-1.43)	1.5 (0.12-1.92)	1.53 (0.57-1.42)	1.1 (0.59-1.79)
	**State Anxiety Inventory (SAI)**								
	< 40 (Low Anxiety)	1	1	1	1	1	1	1	1
	≥ 40 (High Anxiety)	1.03 (0.72-1.47)	1.29 (0.87-1.9)	1.25 (0.85-1.84)	1.41 (0.93-2.13)	1.06 (0.63-1.78)	1.24 (0.8-1.92)	1.28 (0.79-2.06)	1.08 (0.87-1.36)
	**Perceived Stress Scale (PSS)**								
	1st quartile (Very Low)	1	1	1	1	1	1	1	1
	2nd quartile (Low)	0.63 (0.35-1.12)	0.38 (0.19-0.74)	0.67 (0.37-1.22)	**0.36 (0.18-0.71)**	1.20 (0.60-2.42)	1.30 (0.56-2.99)	1.31 (0.62-2.76)	1.31 (0.55-3.09)
	3rd quartile (High)	0.86 (0.49-1.50)	**0.49 (0.26-0.93)**	1.09 (0.61-1.97)	0.54 (0.28-1.05)	0.78 (0.38-1.58)	0.91 (0.39-2.15)	0.99 (0.46-2.13)	1.05 (0.43-2.56)
	4th quartile (Very High)	1.17 (0.61-2.25)	1.35 (0.68-2.69)	1.48 (0.74-2.94)	1.44 (0.70-2.94)	0.84 (0.45-1.57)	1.24 (0.59-2.59)	0.83 (0.42-1.64)	1.27 (0.58-2.80)
2nd Trimester	**General Health Questionnaire**								
	≤ 3 (Low)	1	1	1	1	1	1	1	1
	4–12 (High)	1.17 (0.78-1.76)	**1.67 (1.08-2.58)**	1.32 (0.85-2.06)	**1.76 (1.12-2.76)**	0.97 (0.6-1.59)	1.35 (0.77-2.38)	0.83 (0.49-1.41)	1.29 (0.71-2.35)
	**Trait Anxiety Inventory (TAI)**								
	< 40 (Low Anxiety)	1	1	1	1	1	1	1	1
	≥ 40 (High Anxiety)	1.29 (0.08-1.6)	1.38 (0.5-1.69)	1.45 (0.45-1.74)	1.39 (0.06-1.73)	0.74 (0.74 −1.14)	1.22 (0.32-0.71)	0.7 (0.83-1.11)	1.21 (0.35-1.73)
	**State Anxiety Inventory (SAI)**								
	< 40 (Low Anxiety)	1	1	1	1	1	1	1	1
	≥ 40 (High Anxiety)	1.11 (0.76-1.62)	1.24 (0.82-1.87)	1.22 (0.82-1.81)	1.26 (0.82-1.93)	1.1 (0.69-1.73)	1.51 (0.89-2.55)	1.11 (0.68-1.82)	1.58 (0.91-2.76)
	**Perceived Stress Scale (PSS)**								
	1st quartile (Very Low)	1	1	1	1	1	1	1	1
	2nd quartile (Low)	0.81 (0.52-1.28)	0.74 (0.45-1.22)	0.77 (0.48-1.24)	0.70 (0.42-1.17)	0.62 (0.33-1.19)	0.96 (0.44-2.09)	0.67 (0.34-1.34)	1.08 (0.48-2.43)
	3rd quartile (High)	0.88 (0.54-1.42)	0.71 (0.41-1.22)	0.92 (0.55-1.53)	0.69 (0.39-1.20)	0.81 (0.43-1.55)	0.96 (0.43-2.13)	0.79 (0.40-1.58)	0.92 (0.40-2.11)
	4th quartile (Very High)	1.52 (0.91-2.53)	1.67 (0.97-2.88)	1.60 (0.94-2.72)	1.59 (0.91-2.79)	0.83 (0.42-1.64)	1.77 (0.81-3.90)	0.73 (0.35-1.52)	1.82 (0.79-4.19)
3rd Trimester	**General Health Questionnaire**								
	≤ 3 (Low)	1	1	1	1	1	1	1	1
	4–12 (High)	1.35 (0.9-2.02)	**1.67 (1.08-2.58)**	1.39 (0.91-2.11)	**1.58 (1.01-2.47)**	1.04 (0.62-1.73)	1.35 (0.75-2.43)	0.92 (0.53-1.6)	1.26 (0.68-2.34)
	**Trait Anxiety Inventory (TAI)**								
	< 40 (Low Anxiety)	1	1	1	1	1	1	1	1
	≥ 40 (High Anxiety)	1.11 (0.24-1.44)	1.16 (0.23-1.52)	1.32 (0.08-1.64)	1.25 (0.17-1.62)	0.8 (0.67-1.22)	1.14 (0.39-1.64)	0.92 (0.78-1.61)	1.06 (0.82-1.93)
	**State Anxiety Inventory (SAI)**								
	< 40 (Low Anxiety)	1	1	1	1	1	1	1	1
	≥ 40 (High Anxiety)	1.04 (0.72-1.5)	1.13 (0.76-1.68)	1.24 (0.84-1.82)	1.19 (0.78-1.8)	1.27 (0.8-2.02)	1.37 (0.8-2.34)	1.19 (0.73-1.95)	1.35 (0.77-2.36)
	**Perceived Stress Scale (PSS)**								
	1st quartile (Very Low)	1	1	1	1	1	1	1	1
	2nd quartile (Low)	1.06 (0.66-1.70	0.93 (0.55-1.56)	1.19 (0.73-1.95)	0.84 (0.49-1.43)	1.55 (0.80-2.99)	1.58 (0.72-3.47)	1.90 (0.94-3.85)	1.89 (0.83-4.28)
	3rd quartile (High)	1.14 (0.73-1.80)	1.00 (0.61-1.64)	1.27 (0.79-2.05)	0.92 (0.55-1.54)	0.97 (0.50-1.85)	1.23 (0.56-2.66)	0.96 (0.48-1.93)	1.43 (0.64-3.21)
	4th quartile (Very High)	1.35 (0.82-2.21)	1.25 (0.73-2.13)	**1.72 (1.02-2.91)**	1.20 (0.69-2.10)	0.97 (0.51-1.85)	1.40 (0.66-2.97)	0.81 (0.40-1.63)	1.41 (0.64-3.14)

The association between mental health and GWG. Analyses were adjusted for maternal age, marital status, mothers’ education in years of study, pre-gestational BMI, gestational age, number of rooms per person, alcohol consumption, and serum levels of hemoglobin. Estimated odds ratios (OR) were calculated using an ordinal multinomial logistic regression. OR stands for Odds Ratio; ORa stands for Adjusted Odds Ratio; CI stands for Confidence Interval.

## Discussion

This study investigated the association between maternal mental health and GWG in two Brazilian cohorts, conducted at different periods: the Jundiaí cohort and the Araraquara cohort. We observed that high stress and anxiety levels in the Jundiaí cohort were significantly associated with insufficient GWG, while no significant association was found in the Araraquara cohort. These results suggest that socioeconomic and temporal contexts may influence the relationship between maternal mental health and GWG.

The study results indicate that high levels of stress and anxiety in the Jundiaí cohort are associated with insufficient GWG. These findings support the hypothesis that maternal mental health plays a crucial role in GWG. Previous studies have shown that psychological disorders during pregnancy can negatively affect eating habits and consequently GWG [4,26]. A potential explanation for this is that high levels of stress and anxiety may alter dietary intake, leading to either reduced food consumption due to loss of appetite or emotional eating patterns [44,45]. Additionally, psychosocial stressors, such as financial instability or lack of social support, may contribute to inadequate weight gain in vulnerable groups [45]. However, our findings are specific to the Brazilian context, where the prevalence of mental health problems may be influenced by socioeconomic factors. Studies conducted in Brazil, such as those by Farias et al. (2021) and Gomes et al. (2023), also found associations between maternal mental health and GWG. Our results, however, highlight the variability of these associations in different periods and contexts [8,9] Another study conducted by the BRISA birth cohort, started in 2010 in São Luís, Maranhão, did not show a direct effect of mental disorder symptoms during pregnancy on GWG [46] Although our findings indicate that stress is associated with insufficient GWG, the observed pattern is complex. Specifically, in the Jundiaí cohort, only women in the second quartile of the PSS had significantly lower odds of insufficient GWG. This suggests that moderate stress levels may have a protective effect, possibly by prompting behavioral adaptations such as improved dietary monitoring or increased prenatal care adherence. However, excessive stress may still contribute to inadequate weight gain through metabolic and behavioral pathways. These findings underscore the need for further investigation into how different stress intensities affect pregnancy outcomes.

The lack of significant associations in the Araraquara cohort can be explained by differences in socioeconomic and temporal contexts between the two cohorts. The more recent Araraquara cohort may have benefited from advances in prenatal care practices and greater access to mental health services, which could mitigate the negative effects of stress and anxiety on GWG. Since the late 1990s, Brazil has expanded its maternal healthcare policies, including the introduction of the Rede Cegonha program and increased mental health support in primary care settings. This improved infrastructure may have provided better psychological support and nutritional guidance to pregnant women in the Araraquara cohort, reducing the impact of stress and anxiety on GWG. Additionally, increased awareness of maternal mental health and greater availability of social support interventions could have contributed to the absence of significant associations. Furthermore, socioeconomic improvements and expanded access to essential health services in recent decades may have led to healthier behavioural patterns during pregnancy [47]. Studies conducted in different socioeconomic contexts also indicate that the relationship between mental health and GWG can vary significantly. Moreover, variability in access to health services and available psychosocial support for pregnant women may have impacted the results [48]. Psychosocial support during prenatal care is essential to mitigate the negative effects of stress and anxiety on maternal and neonatal health [6]. The importance of psychosocial support is emphasized in studies showing that targeted interventions can significantly improve health outcomes for pregnant women and their babies [7].

Additionally, the study by Biagio et al. (2024) on domestic violence during the COVID-19 pandemic in the Araraquara cohort highlights the need to consider the influence of external factors and social adversities on pregnant women’s mental well-being. Domestic violence, for instance, was associated with changes in pregnant women’s mental health, suggesting that interventions to improve mental health should consider the context of violence and other social stressors [49].

The relationship between mental health and GWG may be bidirectional. Just as mental health problems can lead to inadequate GWG, inadequate GWG can also cause changes in pregnant women’s mental health. Studies indicate that inadequate GWG can increase the risk of postpartum depression and anxiety [3]. Dissatisfaction with weight gain during pregnancy can lead to feelings of inadequacy and low self-esteem, exacerbating existing mental health problems [29].

Future studies should focus on better understanding the mechanisms by which maternal mental health influences GWG and vice versa. This includes exploring the role of intermediary factors such as eating behavior and social support. Interventions tailored to different socioeconomic and cultural contexts are needed to improve health outcomes for mothers and children. Additionally, it is crucial to investigate the influence of moderating variables such as the level of social support, the quality of prenatal care, and the presence of other environmental stressors in the relationship between mental health and GWG [18].

Existing literature highlights the complexity of the relationship between mental health and GWG. For instance, a study conducted by Farias et al. (2021) found that maternal depression was associated with a higher risk of inadequate GWG, suggesting that different types of mental disorders may have varied impacts on GWG [8]. Moreover, the study by Gomes et al. (2023) indicated that anxiety during pregnancy can lead to caloric restriction and consequently insufficient GWG. These findings reinforce the need for a multifaceted approach in evaluating and intervening in mental health during pregnancy [9].

Variability in results between different cohorts and contexts also suggests that specific factors such as the quality of the social environment and access to mental health resources can mediate the relationship between mental health and GWG. In low-income contexts, where access to mental health services may be limited, the negative effects of stress and anxiety may be exacerbated [4]. This highlights the importance of public policies ensuring equitable access to mental health care for all pregnant women regardless of their socioeconomic context.

In Brazil, the National Health System (SUS) plays a crucial role in providing health care for pregnant women, especially in vulnerable socioeconomic contexts. However, regional disparities and unequal access to mental health services pose significant challenges. Studies have shown that pregnant women in less advantaged regions face greater barriers to accessing adequate mental health care [50]. Moreover, the prevalence of mental health problems may be underestimated due to a lack of appropriate diagnosis and treatment [46].

The findings of this study have important implications for clinical practice and public health policies. Incorporating mental health monitoring and intervention strategies in prenatal care can optimize maternal and neonatal outcomes. Prenatal care programs should include regular mental health assessments and provide appropriate resources and support for pregnant women showing signs of stress, anxiety, or depression [16]. Additionally, training health professionals to identify and manage mental health problems during pregnancy is crucial. Community-based interventions involving education and social support can also be effective in reducing the negative effects of stress and anxiety on GWG. Future studies should explore the effectiveness of different intervention models and identify best practices for supporting maternal mental health in diverse contexts [28].

One of the main strengths of this study is the comparison of two Brazilian cohorts conducted in different periods, allowing a temporal analysis of associations between maternal mental health and GWG. Additionally, the use of advanced analytical methods such as ordinal multinomial logistic regression strengthens the robustness of the results. However, this study also has limitations. Differences in sample size, data collection methods, and cohort retention rates may have influenced the findings. Additionally, unmeasured factors such as dietary quality, social support networks, and regional healthcare variations may have contributed to the observed disparities between the two cohorts.

## Conclusion

Our study identified a significant association between maternal mental health and GWG in the Jundiaí cohort, where higher levels of stress during the first trimester and anxiety in later periods were linked to insufficient GWG. In contrast, no such associations were observed in the more recent Araraquara cohort. This divergence may reflect important temporal and contextual differences, including expanded access to healthcare services, improved prenatal care practices, and greater integration of mental health support through public health initiatives such as SUS and the Rede Cegonha program. These findings underscore the need to consider structural and policy factors when assessing maternal health outcomes over time. They highlight the importance of integrating mental health care into prenatal services, ensuring that psychological support and nutritional counseling are accessible to all pregnant women. Future research should explore the mechanisms behind these associations and assess how evolving social, economic, and healthcare landscapes shape maternal and neonatal health across different populations and time periods.
