# Correction: Mental health and gestational weight gain: A comparison between Brazilian cohorts

**DOI:** 10.1371/journal.pone.0334465

**Published:** 2025-10-13

**Authors:** Audêncio Victor, Maria Paula Carvalho Leitão, Perla Pizzi Argentato, Lívia Patricia Rodrigues Batista, Laisla de França da Silva Teles, Liania A. Luzia, Rinaldo Artes, Patricia H. C. Rondó

There are a number of errors in Table 2. Please see the correct Table 2 here.

**Table 2:** Mental health and its relationship to gestational weight gain in the first, second, and third trimesters in Araraquara and Jundiaí Cohort Studies, São Paulo, Brazil.

Variables		Cohorts studies							
		Jundiai			P values	Araraquara			P values
		Adequate	Excessive	Insufficient		Adequate	Excessive	Insufficient	
1st Trimester	**General Health Questionnaire**								
	≤ 3 (Low)	142 (16.6)	282 (32.9)	161 (18.8)	0.22	50 (10.0)	159 (31.9)	51 (10.2)	0.12
	4 – 12 (High)	54 (6.3)	131 (15.3)	88 (10.2)		44 (8.8)	129 (25.9)	65 (13.1)	
	**Trait Anxiety Inventory (TAI)**								
	< 40 (Low Anxiety)	89 (10.2)	185 (21.2)	111 (12.7)	0.94	50 (8.9)	150 (27.0)	48 (8.6)	0.13
	≥ 40 (High Anxiety)	108 (12.4)	235 (26.9)	144 (16.5)		57(10.3)	169 (30.4)	82 (14.7)	
	**State Anxiety Inventory (SAI)**								
	< 40 (Low Anxiety)	132 (15.1)	279 (31.9)	156 (17.8)	0.31	60 (10.8)	162 (29.1)	71 (12.8)	0.56
	≥ 40 (High Anxiety)	65 (7.5)	141 (16.2)	99 (11.4)		47 (8.4)	157 (28.2)	59 (10.6)	
	**Perceived Stress Scale (PSS)**								
	1st quartile (Very Low)	32 (5.7)	78 (13.8)	52 (9.3)	**0.005**	21 (4.2)	69 (13.8)	23 (4.6)	0.75
	2nd quartile (Low)	39 (6,9)	60 (10.7)	24 (4.3)		19 (3.8)	75 (15.0)	27 (5.4)	
	3rd quartile (High)	40 (7.1)	84 (14.9)	32 (5.7)		20 (4.0)	51 (10.2)	20 (4.0)	
	4th quartile (Very High)	20 (3.6)	57 (10.1)	44 (7.8)		34 (6.8)	94 (18.8)	46 (9.2)	
2nd Trimester	**General Health Questionnaire**								
	≤ 3 (Low)	154 (17.9)	313 (36.5)	171 (19.9)	**0.04**	63 (12.7)	192 (38.8)	67 (13.5)	0.34
	4 – 12 (High)	42 (4.9)	100 (11.7)	78 (9.1)		32 (6.4)	95 (19.2)	46 (9.3)	
	**Trait Anxiety Inventory (TAI)**								
	< 40 (Low Anxiety)	113 (12.9)	214 (24.5)	126(14.8)	0.21	53 (9.5)	182(32.7)	58(10.4)	**0.04**
	≥ 40 (High Anxiety)	84 (9.6)	206 (23.6)	129 (14.8)		54 (9.7)	137 (24.6)	72 (12.9)	
	**State Anxiety Inventory (SAI)**								
	< 40 (Low Anxiety)	144 (16.5)	298 (34.2)	175 (20.1)	0.58	69 (12.4)	199 (35.8)	71 (12.8)	0.22
	≥ 40 (High Anxiety)	53 (6.1)	122 (14.0)	80 (9.2)		38 (6.8)	120 (21.6)	59 (10.6)	
	**Perceived Stress Scale (PSS)**								
	1st quartile (Very Low)	52 (6.1)	110 (12.8)	69 (8.0)	0.07	23 (4.6)	86 (17.4)	24 (4.8)	0.17
	2nd quartile (Low)	64 (7.5)	110 (12.8)	63 (7.3)		27 (5.5)	63 (12.7)	27 (5.5)	
	3rd quartile (High)	47 (5.5)	87 (10.1)	44 (5.1)		25 (5.1)	76 (15.4)	25 (5.1)	
	4th quartile (Very High)	33 (3.8)	106 (12.4)	73 (8.5)		20 (4.0)	62 (12.5)	37 (7.5)	
3rd Trimester	**General Health Questionnaire**								
	≤ 3 (Low)	154 (17.9)	302 (35.2)	171 (19.9)	0.07	64 (13.1)	196 (40.0)	71 (14.5)	0.46
	4 – 12 (High)	42 (4.9)	111 (12.9)	78 (9.1)		28 (5.7)	89 (18.2)	42 (8.6)	
	**Trait Anxiety Inventory (TAI)**								
	< 40 (Low Anxiety)	110 (12.6)	224 (25.6)	133 (15.3)	0.73	61 (8.3)	199 (35.8)	70 (12.5)	0.21
	≥ 40 (High Anxiety)	87 (9.9)	196 (22.5)	122 (13.9)		46 (8.3)	120 (21.6)	60 (10.8)	
	**State Anxiety Inventory (SAI)**								
	< 40 (Low Anxiety)	135 (15.5)	284 (32.6)	168 (19.3)	0.82	72 (12.9)	197 (35.4)	78 (14.0)	0.48
	≥ 40 (High Anxiety)	62 (7.1)	136 (15.6)	87 (9.9)		35 (6.3)	122 (21.9)	52 (9.4)	
	**Perceived Stress Scale (PSS)**								
	1st quartile (Very Low)	60 (6.9)	113 (13.2)	74 (8.6)	0.92	28 (4.6)	79 (17.4)	27 (4.8)	0.66
	2nd quartile (Low)	47 (5.5)	94 (11.0)	54 (6.3)		19 (5.5)	83 (12.7)	29 (5.5)	
	3rd quartile (High)	52 (6.1)	112 (13.1)	64 (7.4)		22 (5.1)	60 (15.4)	26 (5.1)	
	4th quartile (Very High)	37 (4.3)	106 (10.9)	57 (6.6)		23 (4.0)	63 (12.5)	31 (7.5)	
